# When Snoring isn’t just Snoring: Primary Nasopharyngeal Mantle Cell Lymphoma - A Rare Pathology in the Nasopharynx.

**DOI:** 10.51894/001c.7959

**Published:** 2019-07-01

**Authors:** Eytan Keidar, Quynh-Nhu Vu, Carl Shermetaro

**Affiliations:** 1 McLaren Oakland Hospital

**Keywords:** lymphoma, snoring, nasopharyngeal, mantle cell lymphoma

## Abstract

Snoring is a common complaint in the primary care and otolaryngology clinic with a wide differential diagnosis. Primary nasopharyngeal mantle cell lymphoma is a rare cause of a nasopharyngeal mass, which can commonly manifest as snoring. The patient in this case presented with extensive history of recent worsening snoring as well as nasal congestion over the past several months. Additionally, the patient had previously undergone endoscopic sinus surgery several years prior but was lost to follow up. During nasal endoscopy, a nasopharyngeal mass was visualized with near-complete obstruction of the nasal airway. Intraoperative biopsies indicated MCL which is an uncommon pathology presenting in a rare location. Flow cytometry of the biopsy specimen was CD19+, CD20+, CD5+, and positive for lambda light chains with immunohistochemistry showed strong diffuse cyclin D1 nuclear staining on lymphoid cells. PET/CT and bone marrow biopsy were essential in staging disease, predicting success of treatment, and determining optimal treatment planning. Once the diagnosis was established, R-CHOP therapy alternating with R-DHAP for a total of six cycles. This case report highlights the importance of recognizing new or changing symptoms, appropriate diagnostic workup for lymphoma, as well as one of few case reports describing primary nasopharyngeal mantle cell lymphoma.

## Introduction:

Head and neck lymphomas account for 12-15% of all lymphomas and most commonly present as a painless neck mass.^1,2^ Lymphomas are separated into Hodgkin Lymphoma (HL) and Non-Hodgkin Lymphoma (NHL), with NHL being responsible for the majority of cases.^2^ NHL can present itself in lymph nodes or more commonly in extra-nodal sites.^2^ After the gastrointestinal tract, the most common extra-nodal site of NHL is Waldeyer’s ring, which is a series of lymphatic tissue in the upper airway and includes the palatine tonsils, pharyngeal tonsil (adenoid), lingual tonsils, and tubal tonsils of Gerlach. Within Waldeyer’s ring, the palatine tonsils are the most common location (51%; 9% bilateral) followed by the nasopharynx (35%).^3^ Primary Nasopharyngeal Lymphoma (PNL) is not uncommon but only represents approximately 8% of NHL in the head and neck.^4^ The larynx, thyroid, and salivary glands can also be sites of primary NHL.^5^

NHL is further separated between B-cell and T/NK-cell derived lymphomas. B-cell derived neoplasms represent over 80% of NHL.^2^ Mantle cell lymphoma (MCL), a B-cell derived NHL, is an aggressive malignant lymphoma that can be found in extra-nodal sites.^5^ MCL contributes to nearly 6% of all NHL and is largely associated with a poor prognosis. MCL has been rarely described in the literature as a PNL.^6^

To the authors’ knowledge, there have only been two incidences of primary nasopharyngeal MCL described in the literature with this case report being the third.^7,8^ There are several retrospective reviews which separate various types of head and neck lymphomas by location. However, these reports have not reported any incidences of primary nasopharyngeal MCL.^2,3,9–13^ This case report describes a rarely encountered pathology, primary nasopharyngeal MCL.

## Case Report:

The patient was a Caucasian male in his mid-sixties who presented to the authors’ community otolaryngology clinic with chief complaint of nasal congestion over three months. He also admitted to louder snoring recently, with mild clear bilateral rhinorrhea. He denied any hearing loss, epistaxis, cervical lymphadenopathy, or dysphagia. However, he admitted to a history of seasonal allergies.

Several years ago, he underwent functional endoscopic sinus surgery (FESS) with nasal polypectomy, septoplasty, and inferior turbinate reduction to improve his nasal airway and currently has similar symptoms. Several months prior to presentation, he had failed to obtain relief from multiple oral antihistamines, fluticasone, and oxymetazoline. He denied recent imaging or post-surgical follow-up. He admitted to possible sinus infections without improvement after multiple rounds of antibiotics and oral steroid therapy. He reported to having received invasive dental work completed roughly five months prior requiring antibiotic’s due to infection. His other past medical and family history was non-contributory. He admitted to quitting smoking 35 years prior.

**Figure 1:**
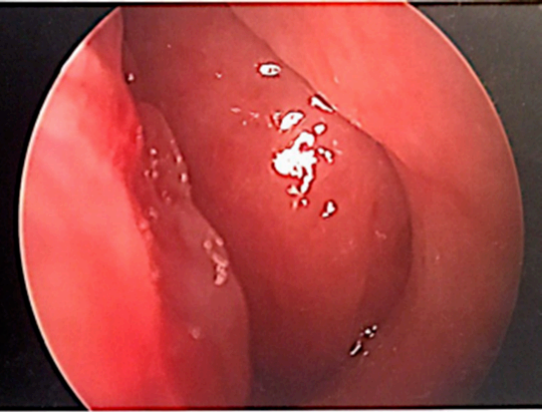
Intraoperative endoscopic visualization of left nasal cavity displaying nasopharyngeal obstructing mass with near complete obstruction of the left nasal airway.

The patient’s physical exam was negative other than a hyponasal voice. Upon nasal endoscopy there were post surgical changes of the maxillary and ethmoid sinuses noted without purulent drainage as well as a large septal perforation. The entire nasopharynx was obstructed with a smooth, fleshy, rubbery mass inhibiting airflow through the nasal cavity (Figure 1).

**Figure 2:**
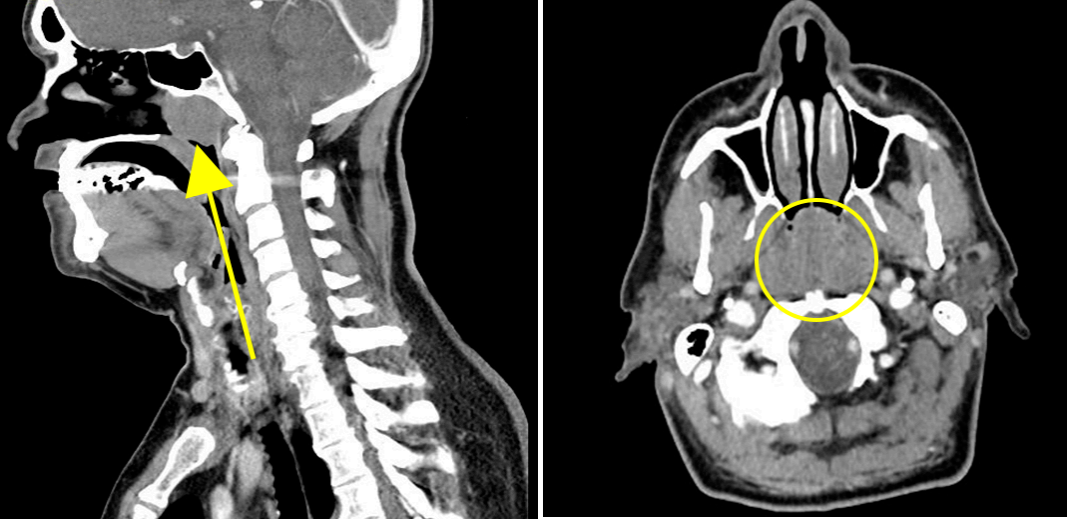
Left: Sagittal CT soft tissue window displaying 3.2cm x 3.0cm nasopharyngeal mass with complete obstruction of nasal airway. Right film shows axial CT with nasopharyngeal mass adjacent to nasal septum.

Tonsils were not enlarged bilaterally. CT sinus revealed a large nasopharyngeal mass obstructing the nasal airway (Figure 2).

A nasopharyngeal biopsy was then performed in the operating room. Initially, a trans-nasal endoscopic biopsy was performed and sent for frozen section as well as in Roswell Park Memorial Institute medium (RPMI). The surgeon was notified that the frozen section was inadequate for definitive diagnosis, advised that squamous cell carcinoma was unlikely, but could not rule out lymphoma. Subsequently, a trans-oral approach (via McIvor mouth gag) was used with a curette into formalin. Flow cytometry was positive for CD19, CD20, CD5, and positive for lambda light chains which are cell surface markers to be able to differentiate between various types of lymphoma as well as target treatment. It was negative for CD3, CD10, and CD23. Immunohistochemistry showed strong diffuse cyclin D1 nuclear staining on lymphoid cells. These pathological markers are commonly seen with MCL.

The patient underwent PET/CT indicating lymph node enlargement with significant uptake at nasopharynx as well as right hilum, which is the root of the lung. Subsequently, a positive bone marrow biopsy was performed followed by treatment with chemotherapy using R-CHOP therapy alternating with R-DHAP for a total of six cycles.

## Discussion:

MCL is a NHL accounting for approximately 6% of all NHL. This condition is due to a t(11;14) translocation in CD5+ B-cells in the mantle zone, which causes over expression of cyclin D1. Cyclin D1, part of a family of D-type cyclins, organizes with cyclin dependent kinases (CDK) to regulate transition of cells from G1 to S phase of the cell cycle. The pathophysiology responsible for the majority of lymphomas involves overexpression of cyclin D1 and even higher in MCL.^6^

More recently, other mutations have been implicated in MCL, which can help providers determine likely treatment outcomes. There are currently two accepted subtypes of MCL. One with mostly SOX11^+^ and unmutated/minimally mutated immunoglobulin heavy chain variable region (IGHV), and the other is SOX11^-^ with mostly mutated IGHV.^14^ MCL is classically found in white males with a median age between 60-70 years old, most commonly presenting at Stage III/IV. Additionally, more recent epidemiological data indicates an even larger disproportion in the incidence of MCL in white older males in the USA. However, the etiology for this gap remains unknown.^9^

MCL, like other lymphomas, is staged using the modified Ann Arbor system which stages lymphomas based on presence and location of disease based on imaging. CT imaging is also involved in diagnosis as well as staging. During evaluation of nasopharyngeal masses, a CT is helpful to distinguish between lymphomas and nasopharyngeal carcinoma. To properly diagnose MCL as well as most lymphomas, the correct type of pathological sections and medium are essential for proper diagnosis. In lymphoma, frozen sections are not sufficient for definitive diagnosis. Ideally, fresh biopsy sections should be sent while ensuring minimal amount of time between biopsy and fixation.^15^ After proper biopsy, additional flow cytometry, immunohistochemistry, and/or genetic studies can be conducted to further classify the lymphoma, which can be beneficial for ideal therapy.

Due to recent genetic and monoclonal antibody research, the classically poor long-term outcomes of MCL have significantly improved.^6^ There is no homogenous therapy for cases of MCL. Although treatment response is high in MCL patients, quick relapse is still an obstacle responsible for its relatively poor prognosis. R-CHOP (rituximab, cyclophosphamide, doxorubicin, vincristine, prednisone) and R-DHAP (rituximab, dexamethasone, HiDAC, and cisplatin) are common in frontline therapy. Additionally, there may be a role for high-dose therapy followed by autologous stem cell rescue (HDT/ASCR) and other single chemotherapeutic agents. Alternatively, the main therapeutic agents for relapsing MCL are Bortezomib, Lenalidomide, and Ibrutinib.^6^

MCL has been described in atypical locations and presentations throughout the head and neck such as hard palate.^16,17^ Although primary nasopharyngeal MCL is a rare finding, it can still present similar to other nasopharyngeal masses. Zhu et al. described a case series on upper aerodigestive tract tumors (ADTT), the majority of which being lymphomas, which included a case of nasopharyngeal MCL.^12^ It was suggested that ADTT, such as primary nasopharyngeal MCL, should be considered in patients with Obstructive Sleep Apnea Syndrome (OSAS) and sudden worsening of symptoms such as gasping or snoring exacerbation.^12^ Due to this patient’s previous history of benign nasal polyposis as well as chronic snoring, the patient stated that he was not initially concerned with his presenting symptoms. The patient’s clinical history such as previous nasal polypectomy, recent dental surgery, as well as allergies endorsed several possible differential diagnoses that may have extended his time to diagnosis.

## Conclusions

This report discusses a rare presentation of MCL discovered as a PNMCL. A thorough clinical history and physical is paramount in identifying MCL, and other ADTT, as well as ensuring a proper endoscopic evaluation and biopsy sample. It is important that primary care as well as otolaryngology clinicians be aware of this type of clinical presentation and rare finding.

### Conflict of Interest

The authors declare no conflict of interest.
